# Early development of *Ensatina eschscholtzii: *an amphibian with a large, yolky egg

**DOI:** 10.1186/2041-9139-1-6

**Published:** 2010-08-03

**Authors:** Andres Collazo, Ray Keller

**Affiliations:** 1 Division of Cell Biology and Genetics, House Ear Institute, 2100 West Third Street, Los Angeles, CA 90057, USA; 2 Department of Biology, University of Virginia, P.O. Box 400328, Charlottesville, VA 22904-4328, USA; 3 University of Southern California, Neuroscience Graduate Program, Hedco Neurosciences Bldg. RM 120E, 3641 Watt Way, Los Angeles, CA. 90089-2520, USA; 4 Department of Cell and Neurobiology, Keck School of Medicine, University of Southern California, CA, USA; 5 Department of Otolaryngology, Keck School of Medicine, University of Southern California, CA, USA

## Abstract

**Background:**

Comparative analyses between amphibians, concentrating on the cellular mechanisms of morphogenesis, reveal a large variability in the early developmental processes that were thought to be conserved during evolution. Increased egg size is one factor that could have a strong effect on early developmental processes such as cleavage pattern and gastrulation. Salamanders of the family Plethodontidae are particularly appropriate for such comparative studies because the species have eggs of varying size, including very large yolky eggs.

**Results:**

In this paper, we describe for the first time the early development (from fertilization through neurulation) of the plethodontid salamander *Ensatina eschscholtzii*. This species has one of the largest eggs known for an amphibian, with a mean ± SD diameter of 6 ± 0.43 mm (range 5.3-6.9; n = 17 eggs). Cleavage is meroblastic until approximately the 16-cell stage (fourth or fifth cleavage). At the beginning of gastrulation, the blastocoel roof is one cell thick, and the dorsal lip of the blastopore forms below the equator of the embryo. The ventral lip of the blastopore forms closer to the vegetal pole, and relatively little involution occurs during gastrulation. Cell migration is visible through the transparent blastocoel roof of the gastrula. At the end of gastrulation, a small archenteron spreading dorsally from the blastopore represents the relatively small and superficial area of the egg where early embryonic axis formation occurs. The resulting pattern is similar to the embryonic disk described for one species of anuran.

**Conclusions:**

Comparisons with the early development of other species of amphibians suggest that an evolutionary increase in egg size can result in predictable changes in the patterns and rate of early development, but mainly within an evolutionary lineage.

## Background

The objective of this study was to investigate how egg size and yolk content affects the mechanisms of morphogenesis in early amphibian development, with an analysis of cleavage, gastrulation and neurulation in a caudate amphibian with a large yolky egg, *Ensatina eschscholtzii*. Gastrulation and early development have been examined in many species of amphibians, but until relatively recently, most studies have considered these events in terms of superficial similarities and differences, rather than in terms of the detailed mechanisms of cell movements [1,2]. Moreover, differences in underlying cellular mechanisms have been overlooked because of the emphasis on a few species that are easy to maintain and manipulate.

The species in which gastrulation and other aspects of early development has been described in the most detail is the frog *Xenopus laevis *[3-7]. Because of this, developmental patterns shown by *Xenopus *are often taken to be general characteristics of Amphibia. However, even some of the earliest aspects of development vary between amphibian taxa. For example, studies on the hylid frog, *Gastrotheca riobambae*, have indicated significant differences in early development from that of *Xenopus*, such as meroblastic and asynchronous early cleavages, an uncoupling of events of the midblastula transition, and blastopore closure before archenteron and notochord elongation [8-10]. All amphibian eggs eventually have holoblastic cleavage (the egg is totally divided by the first and all the following cleavage planes), but in some species (particularly those with large yolky eggs) the first few cleavage planes fail to pass completely through the egg [11-15].

Comparative analysis of the cellular mechanisms of morphogenesis in early development reveals a large variability in ontogenetic processes that were thought to be conserved during evolution. Current studies at the cellular level show that early morphogenesis of amphibians differs in the initial location of prospective tissues and in the types of cellular mechanisms contributing to what appear to be similar morphogenetic movements [1,2]. For example, all urodeles studied thus far [16-19] and most anurans [18,20-22] have prospective mesoderm in the surface epithelial layer of the marginal zone, whereas *Xenopus *has very little [23-25]. In *Xenopus*, multiple cellular processes contribute to involution of the marginal zone, including migration of involuted mesoderm along the blastocoel roof and convergent extension of the involuted mesoderm and noninvoluting neural plate [26,27]. Convergent extension of the mesoderm alone is sufficient to produce involution and many of the other gastrulation movements in *Xenopus *[28-30]. By contrast, migration of mesoderm on the blastocoel roof is needed for gastrulation of the salamander *Pleurodeles *[31,32]. Convergent extension in salamanders appears to occur only in the late gastrula and during neurulation [33-35].

Leaving aside for a moment the question of possible phylogenetic differences in gastrulation between amphibian orders, a major factor potentially responsible for variation in gastrulation mechanisms is difference in egg size. Egg size appears to be largely a function of the amount of yolk in the eggs. Large, yolky eggs may present biomechanical problems to cleavage and gastrulation [36-39], although this has yet to be experimentally demonstrated. A larger egg size in an amphibian would presumably be due to deposition of more yolk in the vegetal region, and in this case, the marginal zone may be displaced animally, near or perhaps above the equator. Under these conditions, convergent extension of the marginal zone would not be useful in closing the blastopore because these movements would squeeze yolk outward [1,2,4].

Salamanders of the family Plethodontidae, such as *Ensatina*, are particularly appropriate for studies of the effects of increased egg size on cleavage pattern and gastrulation because the species have eggs of varying size, including very large yolky eggs [40]. Egg size is related to the evolution of direct development - the development of the embryo into a miniature adult within the egg membranes. This is in contrast to the mode of development among better studied amphibians; that is, those which have free-living aquatic larvae [41]. *Ensatina eschscholtzii *belongs to plethodontid Clade C, and lays one of the largest eggs known for a plethodontid (5 to 7 mm dia in recently oviposited eggs) [40,42,43]. This is only the second species within plethodontid Clade C whose early development has been described in any detail, the first being *Desmognathus aeneus *[44]. Our study of early development in *Ensatina *was undertaken to understand how large egg size influences development and whether salamanders differ from frogs in this respect [45]. We believe that this organism and others like it should be brought into more common use, and thus we also describe the natural history and the methods of obtaining and working with the eggs and embryos.

## Methods

### Obtaining and handling embryos

We gathered material for embryological study by collecting the relatively common adult animals, because egg clutches of *Ensatina *are rarely found in nature. One clutch is laid in the spring of every other year, deep underground or inside large fallen trees [46]. Gravid *E. eschscholtzii *were collected in various California locations in the springs of 1986 to 1989, and oviposition was induced by intraperitoneal injections of 0.1 ml of luteinizing hormone releasing hormone (LHRH; cat. no. L-2761; Sigma-Aldrich, St Louis, MO, USA), diluted to a concentration of 0.05 mg/ml in sterile distilled H_2_O. Females store viable sperm from previous matings for at least 6 months in a spermatheca and the eggs are fertilized as they are laid. In total, 19 egg clutches were obtained, ranging in size from 9 to 16 eggs (mean ± SD 12 ± 2.3). All eggs were raised at 13 to 14°C on wet paper towels inside plastic containers. Because the eggs deposited by non-injected females developed in the same way as those deposited by injected females and because eggs deposited by injected females hatched into apparently normal juveniles, we concluded that the development observed after LHRH-induced oviposition is normal.

### Microscopy, fixation, marking experiments and analysis

Living embryos were examined by light microscopy using a dissecting microscope (Wild/Leica, Heerbrugg, Switzerland), and were then fixed in either 2.5% glutaraldehyde in cacodylate buffer (pH 7.2) or 10% neutral buffered formalin. Fixed embryos were dissected for examination of internal features. Jelly coats were typically mechanically removed with forceps after the embryos were preserved. In some embryos, the jelly coats were removed chemically with 2% cysteine HCL solution (pH 8.1) by placing embryos in this solution for 20 to 50 minutes.

Two of the living embryos were stained with vital dye during the blastula and gastrula stages to test the viability of this technique and to better observe morphogenetic movements. To stain the embryos, most of the jelly coat layers were mechanically removed with forceps, leaving a thin layer of jelly coat and the vitelline membrane. If the vitelline membrane is punctured at these early stages, the embryo explodes through the hole and dies. A crystal of Nile blue sulfate was pressed into the jelly layer and against the vitelline membrane, over the areas to be marked, with a needle or fine forceps. The dye typically soaked through the vitelline membrane within 2 minutes. The regions labeled were recorded and followed over known time intervals.

## Results

### Egg size and developmental rate

**Figure 1:**
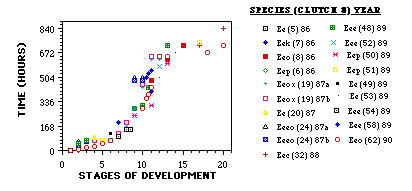
**Rate of early development in *Ensatina eschscholtzii***. The stages of development are from Harrison (1969) and range from 1 (one cell) to 20 (neural folds fused). Figure legends consist of symbol, subspecies (clutch number) year. Key to subspecies of *E eschscholztii*: Eee, E. e. eschscholztii; Eec, E. e. croceator; Eek, E. e. klauberi; Eeo, E. e. oregonensis; Eep, E. e. picta; Eeeo, possible hybrid of E. e. eschscholztii X E. e. oregonensis; Eeox, possible hybrid of E. e. oregonensis X E. e. xanthoptica.

Eggs of *E. eschscholtzii *are very large for an amphibian, with a diameter of 6 ± 0.43 mm (range 5.3 to 6.9; n = 17 eggs) across the recently laid egg within its membranes. This variation in egg size is relatively large for a single species of amphibian [47,48]. Within a given clutch, the variation was substantially smaller. Stebbins observed large variation in ovum diameter in dissected gravid females, with different subspecies having different mean ovum sizes [46]. Eggs are unpigmented, and therefore surface details are difficult to discern. Early development is very slow, with neurulation beginning 23 days after egg deposition at 13 to 14°C (Figure 1). Figure 1 plots the rate of development over stages 1 to 20 for 19 embryos from 17 clutches, with the stage numbers being those used to describe salamander development [49]. Although determining the equivalent stage to those of published normal tables is relatively straightforward at these early stages of development, it is more difficult with postneurula stages because of differences in the relative appearance of superficial structures.

The embryos are easily damaged by mechanical manipulation throughout these early stages. Eggs in which most of the jelly coats are removed and the vitelline membrane is left intact can continue to develop, even at blastula and gastrula stages. However, if the vitelline membrane is removed (possible only if the egg is chemically dejellied first) [5], the flaccid embryo falls apart, presumably from the lack of structural support provided by the vitelline membrane.

### Cleavage pattern

**Figure 2:**
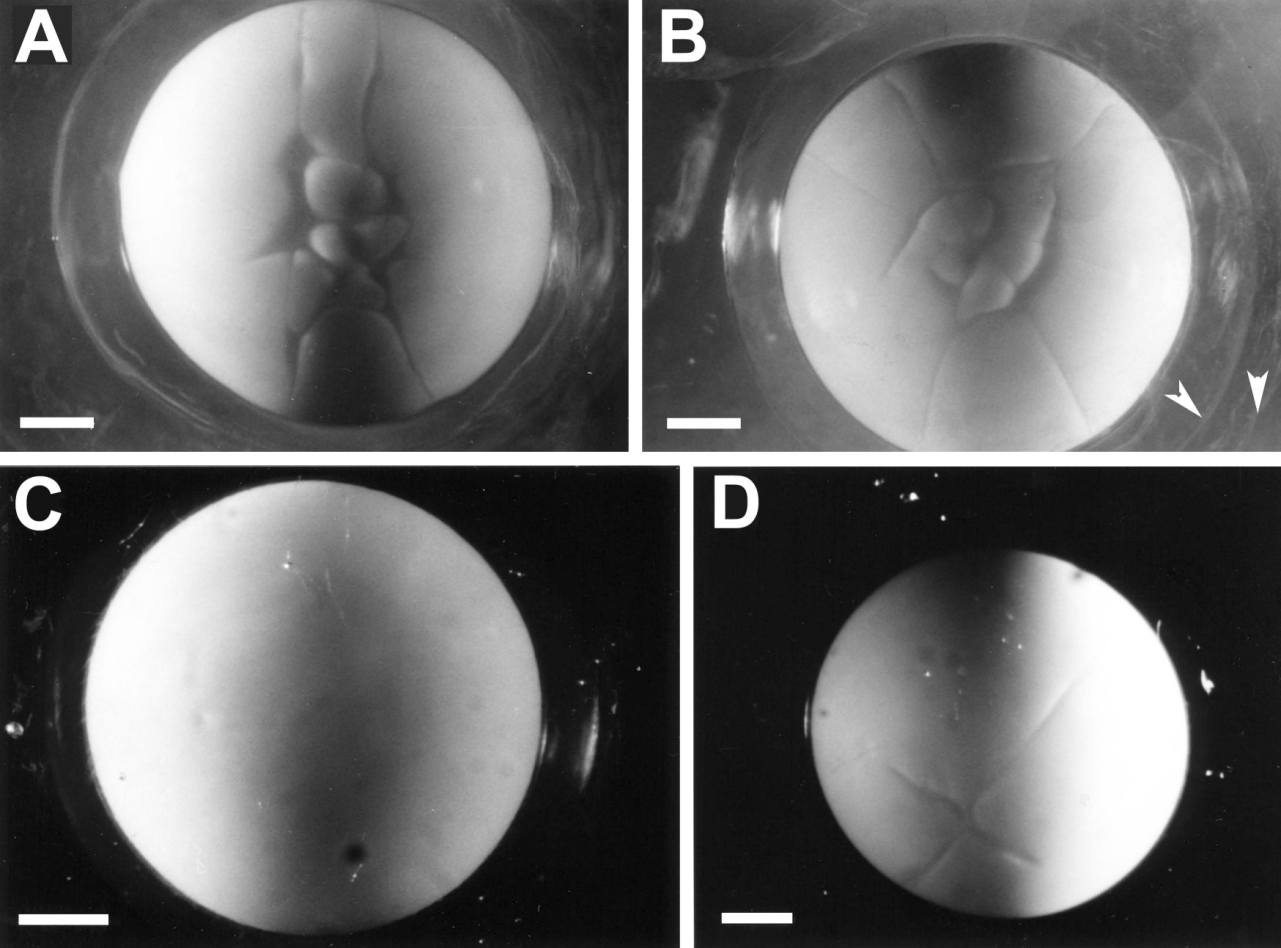
**Animal pole view of four live embryos from two different clutches**. (A, B) Two embryos in early cleavage stages; white arrowheads point to two of the three jelly coat layers. (C) Uncleaved egg; (D) embryo at four cell stage. Scale bars = 1.0 mm.

**Figure 3:**
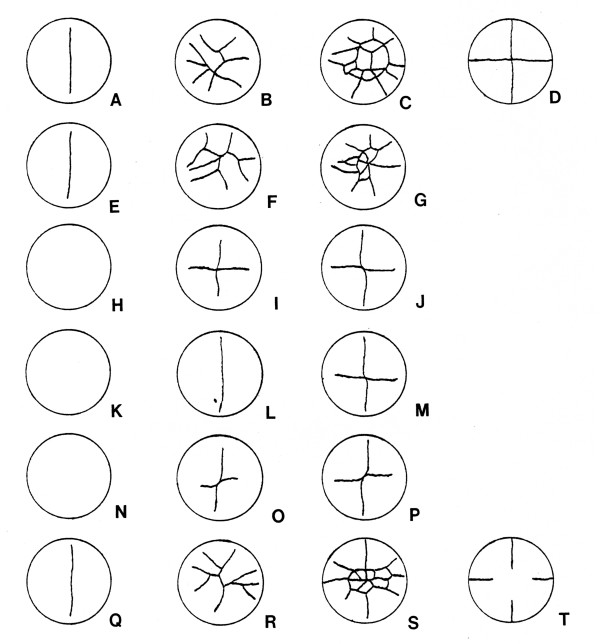
**Early cleavage patterns of same individual eggs at different times**. Six different eggs from the same clutch at three different stages of cleavage. Note the variability in pattern of cleavage. (A-C) Animal pole view of first egg; (D) vegetal pole view of third stage; (E-G) animal pole view of second egg; (H-J) animal pole view of third egg; (K-M) animal pole view of fourth egg; (N-P) animal pole view of fifth egg; (Q-S) animal pole view of sixth egg; (T) vegetal pole view of third stage. Egg diameter is 7.0 mm.

Figure 2 shows photomicrographs of several different embryos at early cleavage stages. Figure 3 shows drawings of the early cleavage pattern at different times for six individual eggs from a different clutch. Cleavage initially occurs only in the animal pole, with no cleavage furrow visible in the vegetal pole until about the 16- cell stage (fourth or fifth cleavage). Cleavages, even if only the animal pole cells are considered, are unsynchronized and uneven after the four-cell (second cleavage) stage. Cleavages that start in the animal pole pass through the entire embryo by the early blastula stages, so cleavage is holoblastic, although it does not appear to be so initially.

### Blastulation and gastrulation

**Figure 4:**
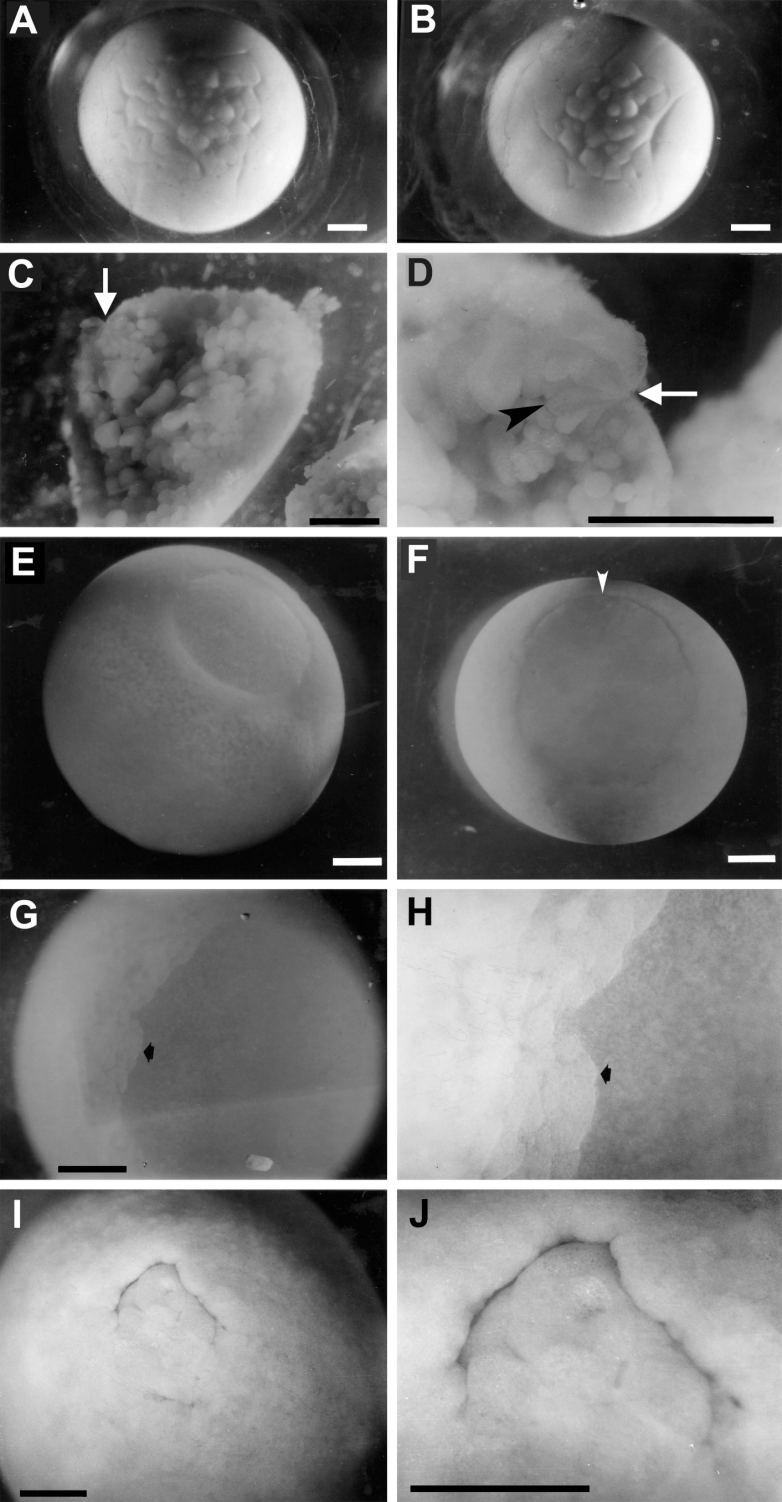
**Developing embryos from early blastula to gastrula stages**. (A, B) Animal pole view of early blastula stages. Two different live embryos from same clutch. (C, D) Two magnifications of same embryo cut in half at the beginning of gastrulation, showing bottle cells. White arrows point to apical end of bottle cells. (C) Lower magnification view that shows large yolky cells near the center of the embryo. (D) High magnification view of the dorsal lip region showing bottle cells. Black arrowhead points to basal edge of one bottle cell. (E, F) Two views of a live embryo at a mid-gastrula stage. (E) Animal pole view showing the blastocoel roof; (F) vegetal pole view of the blastopore. The dorsal side of the blastopore is towards the top (white arrowhead). (G, H) Animal pole view of cells migrating along the blastocoel roof of a live embryo. Arrow points to front of migrating cells. The cells are migrating to the right. (G) low magnification; (H) high magnification. (I, J) Vegetal pole view of the blastopore of a late gastrula stage embryo. Dorsal region is towards the top. (I) low magnification; (J) high magnification. Scale bars = 1.0 mm.

Blastulation is characterized by large asymmetries in cell sizes between animal and vegetal poles. Throughout the blastula stage, the cells of the animal pole are less than half the diameter of those in the vegetal pole (Figure 4A, B). The size differences between the large cells of the vegetal pole and the small cells of the animal pole are abrupt, not gradual. By the beginning of gastrulation, the boundary between the smaller animal cells and the larger vegetal cells lies below the equator of the spherical embryo and marks the area of dorsal lip formation.

By the late blastula stages and throughout gastrulation, the roof of the blastocoel is a translucent epithelial sheet one cell thick. Even at the earliest blastula stages, the roof appears to be just one or two cell(s) thick (Figure 4E). The roof becomes thinner because the majority of cleavages that these cells undergo appear to be lateral as opposed to radial. Epiboly (the spreading of an epithelial sheet of cells) is a major morphogenetic movement during late blastula and gastrula stages, leading to expansion of the thin blastocoel roof.

**Figure 5:**
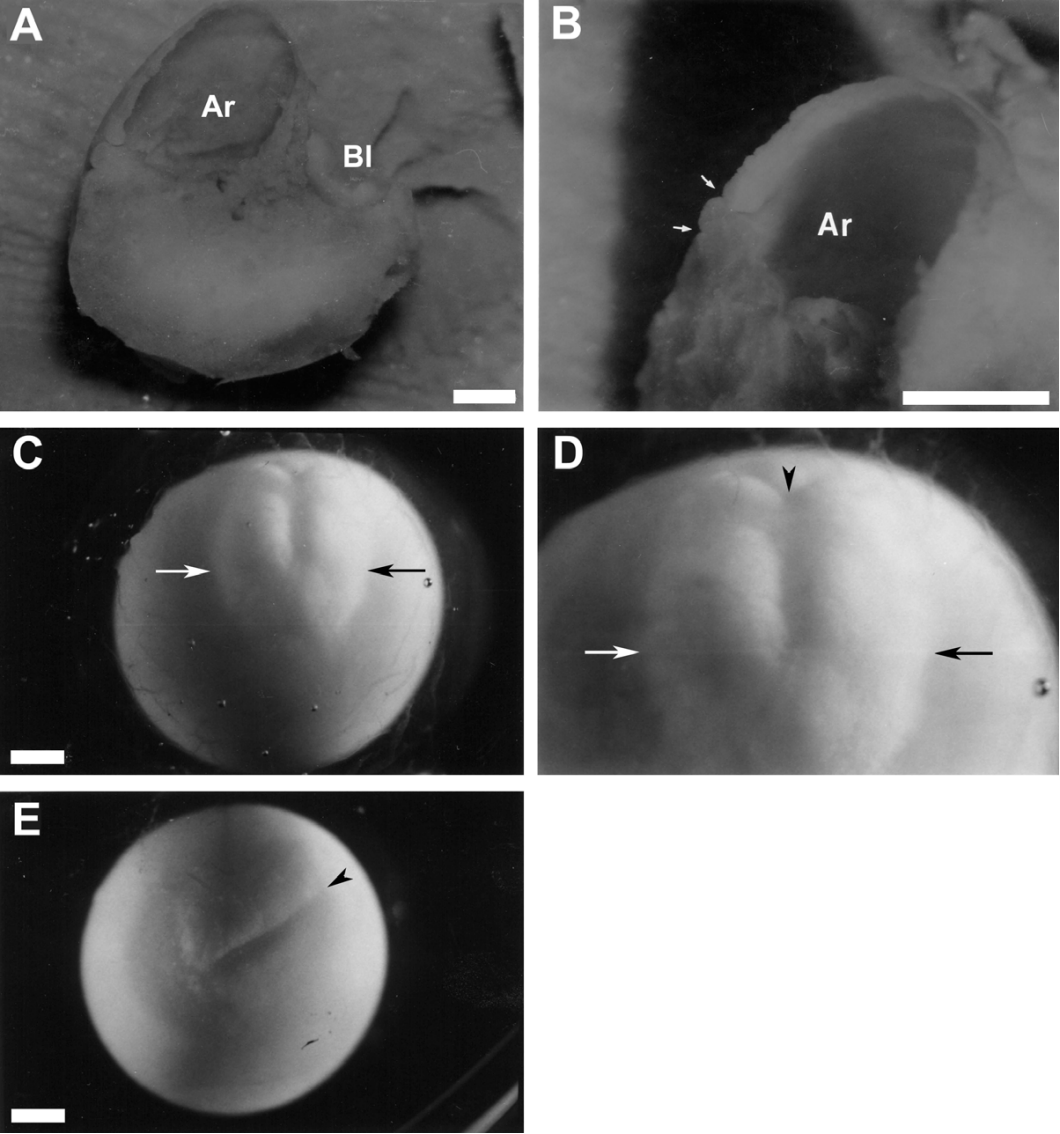
**End of gastrulation through neurula stages showing embryonic disk**. (A, B) Fixed embryo cut in half at the late gastrula stage illustrating internal structures. (A) Low magnification. Dorsal region of embryo is towards the top. (B) High magnification. Arrows delineate the small size of the yolk plug. (C-E) Illustration of the embryonic disk in living *Ensatina *embryos. The two embryos are at an early neurula stage. Arrows indicate the lateral edges of the neural plate. Arrowheads point to closed blastopore. (C) Low magnification view of whole dorsal surface of embryo. Blastopore is towards the top. (D) High magnification of same embryo. (E) Low magnification view of a different embryo with the blastopore toward the top right corner (arrowhead). Ar = Archenteron; Bl = Blastocoel. Scale bar = 1.0 mm.

Gastrulation begins with the formation of bottle cells (Figure 4C, D), as in other amphibians studied [19,30,50-52] but differs in several respects from that of other species. Note the large size of the endodermal cells near the center of the embryo (Figure 4C), even at a stage of development when the morphogenetic movements of gastrulation (for example, epiboly) are beginning. Involuting cells form a large, circular blastopore with relatively little involution in the ventral lip during gastrulation. Because of the relatively small amount of involution, the ventral lip forms close to its final location (Figure 4F). The involuting cells of the dorsal lip can be seen migrating along the thin, (one cell layer thick) blastocoel roof (Figure 4G, H). Closure of the blastopore lip proceeds over the course of 9 days (Figure 4F, I, J) until the yolk plug becomes small. This closure is asymmetrical. A cross-section of the embryo at late gastrula stage (Figure 5A, B) shows the remnant of the blastocoel and the larger archenteron. Note that although the yolk plug is very small, the archenteron is not as large relative to the total egg volume as in *Xenopus *[8,23]. During the completion of gastrulation and the beginning of neurulation, the embryonic disk elongates anteroposteriorly, strongly implying that the cellular mechanism of convergent extension is operative [2,26,28,33,34].

### Formation of an embryonic disk

Gastrulation, neurulation and organogenesis involve a relatively small and superficial part of the total surface of the egg (Figure 5C-E). This pattern of development, although superficially reminiscent of the blastodisc type of development seen in chickens, is actually more similar to the embryonic disk type of development described for *G. riobambae*, for two reasons [9,53]. First, the yolk of chickens remains uncleaved, whereas that of *G. riobambae*, like *Ensatina *and all other amphibians, is cleaved by this stage. Second, most of the embryonic axis (region of organogenesis) forms superficially along the surface of the egg, in the embryonic disk type of development described for some species of amphibians with large eggs. The embryonic disk in *G. riobambae *is derived from a population of yolk-poor cells, symmetrically localized around the small blastopore of the late gastrula, and initially results in a very small and symmetrical archenteron. In *Ensatina*, most of the embryo is derived from a population of yolk-poor cells mostly dorsal to the yolk plug. These cells spread anteriorly as the archenteron elongates. In fixed embryos, it is relatively easy to see which cells are yolk-poor, because such cells are white whereas yolky cells are yellow in color. The early embryonic disk, therefore, takes on a white appearance in the otherwise yellow egg. Formation of an embryonic disk results in a small percentage of egg volume contributing to embryonic structures in both *Ensatina *and *G. riobambae*. However, there are other species of plethodontids in which a small percentage of the egg volume contributes to embryonic structures, but no embryonic disk is apparent [41,44].

### Natural history

**Figure 6:**
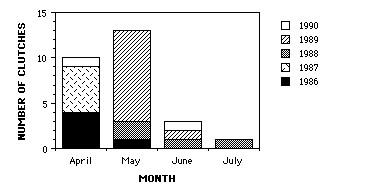
**Bar graph showing months of oviposition of *Ensatina *clutches used in this study**. Accumulated over a period of 5 years.

Months of oviposition over the 5 years of this study are shown in Figure 6. We found that 88% of the females oviposited in April and May, with April being the main month of oviposition in 1986 and 1987, and May in 1989. All the clutches were fertile (at least one embryo developed normally). Approximately 50% of gravid females actually oviposited. The time between injection and oviposition was highly variable. For 16 clutches, the mean was 12.2 days (range 7 to 42.5 days). When the largest clutch size was excluded from the analysis, the mean was 10.2 days (range 7 to 16.5 days). There was no correlation with female body size. Two females oviposited without being injected.

Females turned on their backs to lay eggs, as Stebbins described previously [46]. This unorthodox manner of oviposition has been described in other species of salamanders [54]. Oviposition is slow; more than 24 hours may be required for completion. Previous descriptions of the number of jelly coats (three) and their adhesiveness were confirmed (Figure 2B) [46].

## Discussion

The development of *Ensatina *differs in many respects from that of most other amphibians. We propose that large egg size is the major reason for most of these differences.

### Egg size is a formal evolutionary constraint

An egg can become only so large before a shift from holoblastic to meroblastic cleavage occurs [1,13,55,56]. Such an evolutionary event has occurred at least five times within vertebrates, in lineages leading to hagfishes, elasmobranchs, teleosts, coelacanths and amniotes [40]. The egg size at which the shift from holoblastic to meroblastic cleavages occurs varies between animal taxa; knowing egg size alone does not necessarily predict the type of cleavage. Teleosts and reptiles have meroblastic cleavages, even though some species have eggs the same size as those of amphibians that cleave holoblastically. The identification of a new tissue type in the amphibian embryo - the nutritional endoderm - may provide an intermediate state towards the evolution of amniote meroblastic cleavage [13]. In the large-egged frog, *Eleutherodactylus coqui*, the nutritional endoderm consists of yolk-rich cells that do not contribute to differentiated tissues. It is possible that *Ensatina *also has nutritional endoderm, but further study is necessary. By comparing different groups of amphibians (for example, frogs, salamanders and caecilians) it might be determined if these taxa respond similarly to increases in egg size with regard to early development and gastrulation.

We suggest that the asymmetries and asynchronies in early cleavage are influenced by egg size and represent a formal constraint on evolution [57]. This developmental pattern has been reported in previous studies of other salamanders with relatively large yolky eggs: *Eurycea bislineata *[14], *Desmognathus fuscus *[58] and *Cryptobranchus alleganiensis *[15] (diameters of 2.5, 2.8 and 4.0 mm, respectively). The better studied salamander taxa such as *Pleurodeles*, *Triturus *and the axolotl (*Ambystoma mexicanum*) have smaller eggs (1.7, 2.1 and 1.9 mm diameter, respectively) [59]. *E. bislineata*, *D. fuscus *and *Ensatina *represent two of the three major clades of plethodontids, whereas *Cryptobranchus *is not closely related to plethodontids [42,43]. The fact that the asymmetries and asynchronies in early cleavage seen in these four species are qualitatively similar and that these species represent two disparate salamander families suggests that large egg size and not phylogenetic relationship accounts for the differences in development from amphibians with smaller eggs. *Ensatina*, with the largest egg size of any salamander studied to date, offers the most extreme example of asymmetry and asynchrony. Meroblastic-like cleavages of the sort reported in this study were described in *E. bislineata*, *D. fuscus *and *Cryptobranchus*, but in these species a cleavage plane splits the vegetal pole by about the eight-cell stage, earlier than in *Ensatina*.

Large egg size is clearly a factor in the asymmetrical and asynchronous cleavages seen in *Ensatina*. There may be biomechanical interference with positioning of the mitotic spindles and the actin contractions associated with cytokinesis of the developing embryo [60]. The cellular mechanisms that can cause atypical cleavage patterns have been the subject of both theoretical studies [36,38] and reviews. Most of the theoretical work has concentrated on invertebrate patterns. Experimental studies in sea urchin eggs, using centrifugation to concentrate the yolk and alter the plane of cleavage has provided supporting data for the theoretical work [37]. Centrifugation experiments have also been performed on *Xenopus *to demonstrate the importance of cytoplasmic factors on pattern formation [61,62]. This technique could be used in studies of early cleavage patterns as a means of testing the effect of a concentrated region of yolk on cytokinesis in amphibian embryos.

There is reason to believe that the mechanisms that govern cleavage in invertebrates may have some relevance to the understanding of amphibian cleavage [60]. Work on invertebrates can help direct research that investigates mechanisms for the production of atypical cleavage patterns in amphibians. For example, a potential factor causing asymmetrical cleavage, not directly related to yolk, is an attachment of the mitotic spindle to the animal cortex of the dorsal region as seen in the mollusc *Spisula solidissima *[63]. Studies have demonstrated that cortical complexes are important for pattern formation in *Xenopus *[64-66].

### Blastulation

Blastula formation in *Ensatina *appears to be a modification of the typical amphibian pattern and again the differences can probably be attributed to the large amount of yolk. The blastocoel, compared with the diameter of the egg, is much smaller in *Ensatina *than in smaller-egged amphibians. Large and yolky endodermal cells make up most of the egg, which means that the animal pole cells represent a smaller proportion of the egg, creating a relatively small blastocoel. A one-cell-thick blastocoel roof means that the cells that form tight junctions to the outer environment of the embryo are also the cells secreting an extracellular matrix on which involuting cells migrate [27]. In *Xenopus*, the blastocoel roof is several layers thick and consists of two different, stratified populations in terms of protein secretion [67]. This implies that the epithelial sheet of the blastocoel roof in *Ensatina *has a single cell population that exhibits the role of two cell populations in other species, assuming similar secretory behavior to that in *Xenopus*. The thin (one cell thick) blastocoel roof is a characteristic of other species of plethodontids, some with relatively small eggs [40].

### Convergent extension versus cell migration

Convergent extension of the involuting cells during gastrulation is the major mechanism moving cells along the blastocoel roof in *Xenopus *[26,28,68,69]. Migration (the crawling of the cells along the blastocoel roof) is another possible cellular mechanism and is the major, although not exclusive, mechanism of involution in the salamander *Pleurodeles waltl *[31,32]. The observations in these two species have been used to argue that cell migration is the more important morphogenetic process during gastrulation in salamanders, whereas convergent extension is the more important morphogenetic process in frogs [32,70]. Interestingly, more recent analyses of other species of Anurans suggests that there are frog species without convergent extension [2]. The importance of the role of convergent extension during salamander neurulation is accepted [33,34]; the controversy here is its role during gastrulation. In the salamander *A. mexicanum*, both migration and convergent extension are important mechanisms [2,16]. Convergent extension may be occurring during *Ensatina *gastrulation because Anuran species without convergent extension have symmetrically closing blastopores, whereas *Ensatina *has an asymmetrically closing blastopore with more involution on the dorsal side. Of course, in other species of salamanders, subduction, involving apical constrictions and ingression of mesoderm, seems to be a major morphogenetic process during gastrulation and the same may well be true for *Ensatina *[2,19]. This study could not distinguish which mechanism of involution was more important or even estimate the extent of ingression in this species, but it did demonstrate that cell migration occurs along the blastocoel roof of *Ensatina*.

### Blastopore closure and archenteron formation

Blastopore closure is a biomechanical problem when the egg is as large as in *Ensatina *[4,26]. The slowness of the gastrulation process is one consequence. The differences in the rate of blastopore closure between *Ensatina *and other salamanders are probably due to the larger absolute egg size (including amount of yolk) of *Ensatina*. Epiboly of the noninvoluting cells presumably helps surround the yolky endoderm cells. The vitelline membrane is very elastic and tightly stretched around the embryo, and it provides support for the flaccid *Ensatina *embryo. Without this support the embryo could not develop, much less gastrulate. These differences do not entail any new cellular mechanisms.

Keller predicted that, unless there is some fundamental change in gastrulation mechanisms, the dorsal lip of a large-egged amphibian must form below the equator in order for blastopore closure to occur, which seems to borne out in the case of *Ensatina *[4]. By contrast, the yolky sturgeon egg appears to avoid this difficulty by reproportioning and retiming these movements so that extension occurs first, without convergence, moving the marginal zone below the equator and then converging to close the blastopore [26,71]. Despite their apparent versatility, convergent extension movements are not universally used in gastrulation, even for relatively small eggs, where they offer few apparent disadvantages. For example, *P. waltl*, which does not appear to use convergent extension until later stages, has eggs not much larger (1.7 mm) [72] than those of *Xenopus *(1.4-1.5 mm) [3], and their marginal zone lies near the vegetal pole. Subduction, the morphogenetic mechanism for blastopore closure seen in many salamander species (including *P. waltl*), can also result in asymmetrical blastopore closure [2,19]. Even other species of frogs, some with larger eggs, do not seem to use convergent extension during gastrulation, as shown by the lack of notochord elongation as visualized by Brachyury staining [2,8]. It is clear that there is still much to understand about the effect of egg size on morphogenesis.

The archenteron that is formed at an advanced stage of blastopore closure is small in *Ensatina *(Figure 5). The formation of such a small archenteron is indicative of the formation of an embryonic disk, as described in the tropical tree frog *Gastrotheca. riobambae *(Anura: Hylidae) [8,73]. However, the archenteron of *Ensatina *is larger than that of *G. riobambae*. Furthermore, it is not symmetrically located around the blastopore but is skewed almost totally towards the dorsal side. Interestingly, this aspect of archenteron formation is more similar to that observed in *Xenopus *than to that observed in Dendrobatid frogs and *G. riobambae *[8]. Archenteron formation in *Ensatina *is similar to that in another large-egged salamander, *Andrias japonicus *[74].

### Comparisons with other amphibian clades

Early development of large amphibian eggs has rarely been examined in more than a cursory manner, and few reports are available for comparison with our own observations. The third order of amphibians, the Gymnophiona, has some species with large eggs and many of the developmental characteristics seen in *Ensatina*: initially incomplete cleavage, and presence of a thin blastocoel roof and an embryonic disk [11,75]. However, early developmental data on gymnophiones are very scarce.

Early development of anurans is known in more detail and in more species. *G. riobambae *shows the three patterns of early development mentioned for gymnophiones and these patterns are similar in appearance to those observed in this study: initially incomplete, asynchronous and asymmetrical cleavage; a one-cell-thick, translucent blastocoel roof; and an embryo forming from a superficial embryonic disk [9,53]. In *G. riobambae*, large egg size has been implicated as a possible explanation for these developmental patterns [10].

The above discussion of the possible effects of large egg size on early development has been cast in a causal light, concentrating on mechanistic explanations. In discussing the effects of large egg size on early development in an evolutionary context, it must be understood what is meant by large egg size. There are at least two possible criteria for defining what is a large egg size for amphibians: (i) an absolute measure based on the range of egg sizes (generally diameter) in all species of a taxon; or (ii) a relative measure based on a ratio of egg size (such as indicated by cytoplasmic volume) to some developmentally crucial cell parameter such as cytoskeletal elements and/or nuclear volume (typically represented by genome size) [76,77]. A large egg size as defined by the first criterion will have implications to related quantitative characters such as volume, surface area and mass. We believe it is not unreasonable to assume some correlation between these measurements and another quantitative measure, the amount of yolk. A large egg size as defined by the second criterion is more complicated and probably more realistic. However, there are probably interspecies comparisons where the first criterion is a close approximation of the underlying cellular differences. Any discussion on the effects of large egg size on early development should take these criteria into account.

Both *G. riobambae *and *Ensatina *could be considered to have a large egg size by the usage of either criterion. *Ensatina *has a large egg size in an absolute sense because the egg diameter is close to the maximum observed across all amphibians (this maximum is 10 mm for urodeles and anurans [55], slightly more for gymnophiones). The absolute egg diameter of *G. riobambae *is not small, but it is smaller (4-5 mm diameter) than that of *Ensatina*. However, by taking into account genome size (the only relative and crucial cell parameter for which data are broadly available), the eggs of *G. riobambae *have a larger effective egg size because of their much smaller genome size [9,78]. If both species are considered to have a large egg size, because they fulfill either criterion, then the unusual patterns of early development observed in both species (initially incomplete, asynchronous and asymmetrical cleavage; a one-cell-thick translucent blastocoel roof; and an embryo forming from a superficial embryonic disk) correlate with large egg size.

Data on egg size and early development of some other species of amphibians contradict this simple correlation, and suggest a more complicated evolutionary interaction between large egg size (defined by either of the two criteria mentioned) and morphogenetic patterns of early development. Studies on the development of a frog from a different family (*Eleutherodactylus coqui*; Leptodactylidae) supports this conclusion [55,79]. The egg diameter of this species is the same as *G. riobambae*, yet its development is not nearly as unusual as that of *G. riobambae*; for example, *E. coqui *does not develop a superficial embryonic disk [55,79]. Because genome sizes are similar for these two genera, determination of which has a larger egg size based on the relative criteria does not alleviate this apparent contradiction. Embryos of *E. coqui *do have one unusual feature in their early development, a translucent blastocoel roof, which indicates thinness [79].

Using the terminology of Gould, we propose that the differences between these two species of anurans suggest that the effect of egg size on two of the three developmental patterns discussed (one-cell-thick blastocoel roof and an embryo forming from a superficial embryonic disk) are not a formal but possibly a historical constraint in frogs [57]. This is in contrast to effect of egg size on early cleavage patterns, which, as stated above, appears to be a formal constraint. If large egg size always correlated with unusual patterns of early development, then egg size could be considered a formal constraint, but this is not the case because frogs with similar sized eggs can have very different developmental patterns.

### Comparisons with other species of plethodontids

A related plethodontid, *Batrachoseps*, shows two of the three patterns of early development seen in *Ensatina *and *G. riobambae *(initially incomplete, asynchronous and asymmetrical cleavage and an embryo forming from a superficial embryonic disk), but it differs in having a blastocoel roof two to three cells thick [80]. Other species of plethodontids, such as *Desmognathus quadramaculatus *and *D. wrighti*, show all three patterns of early development [40,41,44]. *Batrachoseps *has a genome size similar to that of *Ensatina *[78] but an egg diameter (3.4 mm) just over half that of *Ensatina*; nevertheless, *Batrachoseps *is considered to have a large, yolky egg relative to that of other amphibians. The two species of *Desmognathus *have the same genome size [81], which is less than half that of *Ensatina*, and differ in egg size, with *D. quadramaculatus *having a larger egg diameter (3.9 mm) than that of *D. wrighti *(2.3 mm) [40]. It is possible that the two species of *Desmognathus *have a large egg size based on a relative criteria using a ratio of genome size to egg diameter, explaining their unusual early development [41,44]. Further studies need to be carried out on the early development of other species of plethodontids to better understand the extent of variation in early development

In interspecies comparisons, both between anuran families and within plethodontids, the effects of increased egg size on patterns of early development appear complex. The species comparisons that do not support a simple correlation between large egg size and patterns of early development are those between distantly related taxa, suggesting that large egg size may be a useful predictive factor for early development only within an evolutionary lineage.

### Factors influencing the rate of development

In most species of salamanders studied, as in this one, development is slow, but in salamanders this may be due not only to egg size but also to the large amount of nuclear DNA, which is known to slow cell cycle time and regeneration [82,83]. Comparative studies between anurans have led researchers to conclude that large egg size does not necessarily slow the rate of development [10]. Based on comparative studies of plethodontid development, genome and egg sizes seem to affect the rate of early development only when they reach extremely large sizes (Collazo and Wake, unpublished data). The differences in genome sizes necessary to affect the rate of early development may need to be larger than those seen in the four species of plethodontids discussed above. However, the relatively large size of the genome in *Ensatina *together with its large egg size may be acting synergistically to slow down the rate of development. We believe that the most important single factor for the slow rate of development in *Ensatina *is probably egg size, simply because of the mechanics of cytokinesis through such a large egg volume and morphogenesis across such a large surface area. The same situation seems to be true in anuran lineages. Even though the genome size of *G. riobambae *is larger than that of most other hylids (6 pg per haploid genome), it is not much larger, and is in fact small relative to that of salamanders and many other anurans [9,78,84]. We see no obvious correlation between large genome size and the unusual patterns of development in the amphibians we have discussed. Further studies of the potential role of large genome sizes on early development are warranted.

## Conclusions

We suggest that large egg size and the large amount of yolk associated with this increase explains the unusual patterns of early cleavage in a biomechanical context, and the unusual patterns of blastulation and gastrulation in a morphogenetic context. Comparison with frogs having different amounts of yolk supports this, although the interspecies comparisons do suggest a caution in that the role of increased egg size on early development requires a phylogenetic context. Evolutionary effects of large egg size on the rate of development are similar to that of large genome size in that large increases are necessary to produce a slower rate of development. Experiments could be performed to corroborate the importance of yolk; these would involve changing of egg size by removing and adding yolk. Manipulations of the amount of yolk are very difficult in *Ensatina*, although manipulations have been successfully performed on groups as diverse as *Sceloporus *lizards and sea urchins [85,86]. Fortunately, *Ensatina *has seven subspecies, some of which vary greatly in size [87,88]. Our data include the two most extreme subspecies in terms of egg diameter. Stebbins measured the diameter of ova in gravid females and found that *E. eschscholtzii picta *had the smallest mean egg size whereas *E. eschscholtzii croceater *had the largest [46]. Preliminary results suggest that females of the smaller subspecies lay smaller eggs whose postneurula development is more rapid than that of the larger eggs used in this study. These 'natural' experiments provide an opportunity to study the effects of egg size on development.

Large egg size in amphibians appears to produce significant and predictable changes in early development, even across different orders. The criteria used to define large egg size must be clearly stated. A phylogenetic context is also important in further understanding the influence off egg size on morphogenesis during early development and on the rate of early development.

## Competing interests

The authors declare that they have no competing interests.

## Authors' contributions

AC collected and analyzed the embryological material, and discussed and drafted the manuscript. RK helped in the fixation and dissection of embryos to reveal and identify internal structures. RK also provided the intellectual background for interpreting early development and helped draft the manuscript.
